# Loss of Pol32 in *Drosophila melanogaster* Causes Chromosome Instability and Suppresses Variegation

**DOI:** 10.1371/journal.pone.0120859

**Published:** 2015-03-31

**Authors:** Patrizia Tritto, Valeria Palumbo, Lucia Micale, Marco Marzulli, Maria Pia Bozzetti, Valeria Specchia, Gioacchino Palumbo, Sergio Pimpinelli, Maria Berloco

**Affiliations:** 1 Dipartimento di Biologia, Università degli Studi di Bari “Aldo Moro”, 70125 Bari, Italy; 2 Dipartimento di Biologia e Biotecnologie “C. Darwin”, Università degli Studi di Roma “La Sapienza”, 00185 Roma, Italy; 3 IRCCS Casa Sollievo Della Sofferenza Hospital, 71013 San Giovanni Rotondo, Italy; 4 Department of Microbiology and Molecular Genetics, University of Pittsburgh School of Medicine, Pittsburgh, PA 15219, United States of America; 5 Dipartimento di Scienze e Tecnologie Biologiche ed Ambientali, Università del Salento, 73100 Lecce, Italy; 6 Istituto Pasteur—Fondazione Cenci Bolognetti and Dipartimento di Biologia e Biotecnologie “C. Darwin”, Università degli Studi di Roma “La Sapienza”, 00185 Roma, Italy; St. Georges University of London, UNITED KINGDOM

## Abstract

Pol32 is an accessory subunit of the replicative DNA Polymerase δ and of the translesion Polymerase ζ. Pol32 is involved in DNA replication, recombination and repair. Pol32’s participation in high- and low-fidelity processes, together with the phenotypes arising from its disruption, imply multiple roles for this subunit within eukaryotic cells, not all of which have been fully elucidated. Using *pol32* null mutants and two partial loss-of-function alleles *pol32^rd1^* and *pol32^rds^* in *Drosophila melanogaster*, we show that Pol32 plays an essential role in promoting genome stability. Pol32 is essential to ensure DNA replication in early embryogenesis and it participates in the repair of mitotic chromosome breakage. In addition we found that *pol32* mutantssuppress position effect variegation, suggesting a role for Pol32 in chromatin architecture.

## Introduction

A eukaryotic chromosome is a highly organized DNA-nucleoprotein complex that regulates its metabolism—transcription, replication, recombination and repair—by intricate and continuous modifications of its protein components. The accuracy of genome replication is ensured by high-fidelity DNA polymerases (Pols). However, if DNA damage prevents these high-fidelity polymerases from continuing DNA replication, the cell tries to overcome the obstacle with a damage tolerant pathway, using specialized translesion Pols that lack exonucleolytic proof-reading activity. With the aid of these Pols, DNA synthesis can proceed without the collapse of the replication fork, but with the risk of possible mutations from incorrect nucleotide incorporation.

Pol32 is a small accessory subunit of both DNA Polymerase δ (Polδ) [1] and Polymerase ζ (Polζ) [2–4]. It is conserved during evolution, and participates in both high- and low-fidelity repair processes [5].

Polδ is a high-fidelity polymerase essential for chromosome replication, recombination and DNA repair in eukaryotic cells [6]. What is currently known about the structure and functions of Polδ comes mainly from studies on *Saccharomyces cerevisiae*, *Schizosaccharomyces pombe*, and humans.

In the budding yeast *S*. *cerevisiae*, Polδ is composed of three subunits, the catalytic subunit Pol3 and the Pol31 subunit, both essential, and a third small accessory subunit, Pol32 [7]. In the fission yeast *S*. *pombe*, Polδ contains four subunits: the catalytic subunit Pol3, and three small accessory subunits Cdc1, Cdc27 (corresponding to Pol31 and Pol32 respectively), and Cdm1 [8]. Orthologs of all the four yeast proteins, named p125, p50, p66 and p12 respectively, form mammalian Polδ [9].

In *Drosophila melanogaster*, Polδ is a three-subunit complex: the catalytic subunit DmPolδ, the putative Pol31 ortholog encoded by *CG12018*, and the recently identified small subunit Pol32; no Cdm1 ortholog has been identified so far [10–13]. *Drosophila* Pol32 contains conserved Polα and PCNA (proliferating cell nuclear antigen) interacting domains [14].

Pol32 is dispensable for growth in budding yeast, whereas the orthologous Cdc27 is an essential protein in fission yeast. Pol32 has two basic functions: to enhance Polδ complex activity during replication, and to repair DNA. Cells lacking Pol32 display defects in replication with frequent stalls, as well as an increased sensitivity to hydroxy-urea (HU), ultraviolet (UV) radiation and methylmethane sulphonate (MMS) mutagens; moreover, mutants display defects in break-induced replication (BIR) [15–17]. Pol32 is also required for telomerase-independent telomere maintenance [18].

In *D*. *melanogaster*, Pol32 is required for the repair of DNA Double-Strand Breaks (DSBs) by homologous recombination (HR) involving extensive DNA synthesis [13]. DSB lesions can arise during endogenous metabolism or they can be induced from exogenous sources such as ionizing radiation (IR) or chemical agents. If not properly repaired, DSBs lead to chromosome loss or chromosome rearrangements [19]. HR is one of the strategies that allow cells to repair DSBs [20].

In Yeast, Pol32 is also required for Polζ-mediated translesion synthesis (TLS) [17]. Polζ is not an error-prone Pol *per se*, but it extends DNA from mispaired bases after an incorrect nucleotide insertion by a Y-family Pol, across a replication-blocking lesion [21]. Yeast Polζ is composed of the catalytic subunit Rev3 and the accessory Rev7 subunit [22]; recent work demonstrates that both Pol32 and Pol31 are also functional subunits of Polζ [2–4].

Polζ is not essential for yeast viability, but a deletion of Rev3 results in a marked reduction in the frequency of base pair substitutions and frame shift mutations after UV radiation or chemical treatment with DNA damaging agents [3,23]. In mice, besides its role in TLS, a disruption of Polζ leads to embryonic lethality [24].

DmREV3 and DmREV7 subunits form *D*. *melanogaster* Polζ (DmPolζ) [25]. *rev3* mutants are sensitive to MMS, nitrogen mustard and ionizing radiation; DmPolζ plays an important role in HR repair [13]. A biochemical interaction between DmREV7 and Pol32 has been predicted in a protein interaction map (DPiM) [12,26].

*Drosophila* is a good model for the characterization of genes involved in DNA repair and genome stability. In addition, although gene functions are evolutionarily conserved, some mutations that are lethal in higher organisms, such as Polδ and Polζ are in mice [24,27], are viable in *Drosophila*.

In this study, we investigated previously uncharacterized aspects of the role of Pol32 in promoting genome stability. Our results support the identification of CG3975 as the *pol32* ortholog, as Kane and collaborators advocate in their paper [13].

We induced new mutant alleles of *pol32*; by analyzing the mutant phenotypes we found that Pol32 is required to ensure DNA replication during the early embryonic nuclear divisions. In addition we showed that the loss of Pol32 prevents the repair of DNA breaks in mitotic brain cells, conferring sensitivity to IR, and is important for EMS and ENU-induced damage repair. Moreover we found that *pol32* mutants suppress position effect variegation, suggesting a novel role for Pol32 in the dynamics of chromatin architecture.

## Materials and Methods

### *Drosophila* chromosomes and culture conditions

*Drosophila* strains and crosses were raised at 25° on standard cornmeal yeast agar medium. Genetic markers and strains are described in [28] and FlyBase (http://flybase.bio.indiana.edu). The stocks used in the present work were supplied by Bloomington Drosophila Stock Center unless otherwise noted. The wild-type *Oregon-R* strain was used as a control unless otherwise noted.

**Fig 1:**
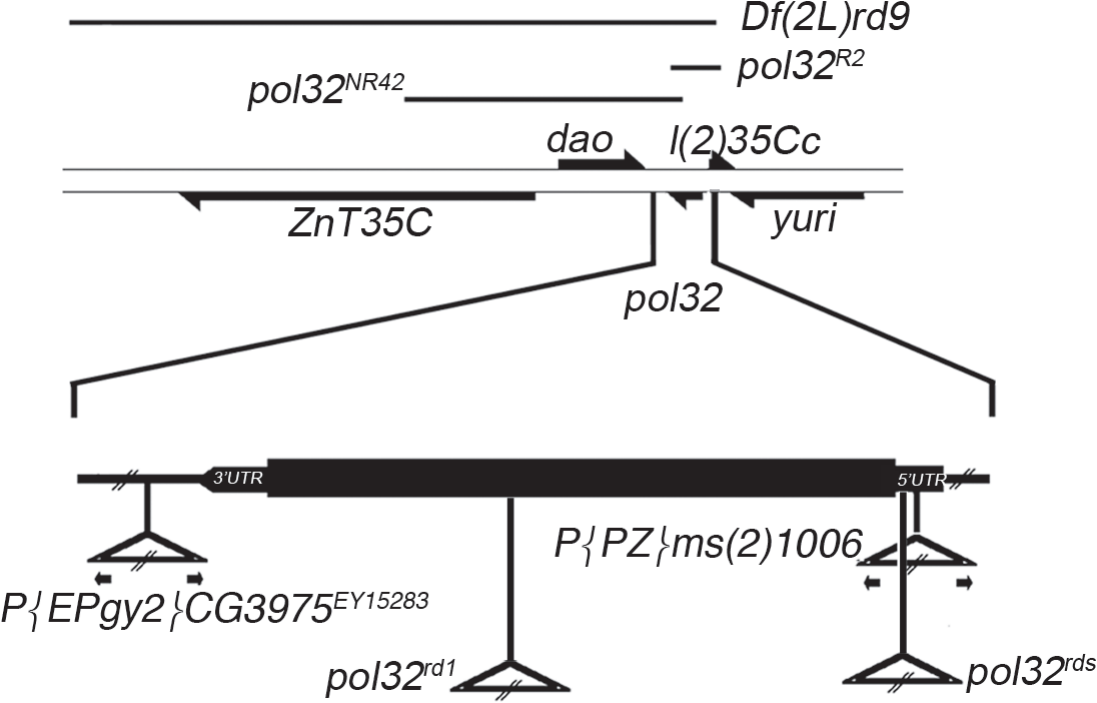
Diagram of *pol32* genomic region. Genes are depicted as arrows indicating the direction of transcription; the thick lines represent the coding region and the thinner lines, the intergenic regions. The lines above indicate the extent of the deletions used. Below, in the enlargement of *pol32* gene, triangles indicate the locations of the transposable element insertions.

*P{PZ}ms(2)1006* ([29], kindly provided) has a single *P{PZ}* insertion in the 35B2-C polytene region carrying *rosy* (*ry)* as a marker gene. By inverse PCR, the insertion was mapped in the 5’ UTR region, 17 bases upstream of the *pol32*, ATG site (Fig. 1). *P{EPgy2}CG3975* ^*EY15283*^ [30] is reported as a single P insertion carrying *white (w)* as a marker gene, inserted downstream of 3'UTR of CG3975 (2L:15,254,643) [31], (named *pol32* in [13]), (Fig. 1). Chromosome rd^1^ carried a lethal mutation which was removed by recombination, and an unidentified female sterile mutation. The recombinant rd^1^ chromosome was used in the present work. To avoid interference with the female-sterile and likely occurring other second site mutations on the second chromosome, trans-heterozygotes with *Df(2L)pol32* ^*R2*^ and *Df(2L)pol32* ^*NR42*^ (recovered in the present work, henceforth designated *pol32* ^*R2*^ and *pol32* ^*NR42*^, respectively) were used in all experiments. A *mei-W68* ^*k05603*^/*Cy* stock was used to construct the recombinant double mutant *pol32* ^*NR42*^ *mei-W68/Cy*, used for the embryogenesis analysis.

### P-element mobilization and molecular characterization of excision lines

We recovered loss-of-function alleles by imprecise excision of *P{PZ} ms(2)1006* and *P{EPgy2}CG3975* ^*EY15283*^ insertions by standard crosses using a Δ 2–3 transposase source [32]; the excisions were identified by following loss of *ry* or *w* expression, respectively. Polymerase chain reaction (PCR) was used to screen for the presence of small deletions flanking genomic sequences caused by imprecise excision of P-elements. The alleles *pol32* ^*R2*^ and *pol32* ^*NR42*^, recovered from *P{PZ}ms(2)1006* and *P{EPgy2}CG3975* ^*EY15283*^ respectively, were used for subsequent genetic and molecular analysis. The genomic regions flanking the *P{PZ}ms(2)1006* and *P{EPgy2}CG3975* ^*EY15283*^ insertions were recovered by plasmid rescue according to [33], cloned into Bluescript (Stratagene, La Jolla, CA), and sequenced. The primers used for molecular characterization of the *P{PZ}ms(2)1006* and *P{EPgy2}CG3975* ^*EY15283*^ insertions and for the excision lines were: **Plac1** 5’-GAA GCCGATAGCTGCCCTG-3’; **UB** 5’-AACGCGGCCGCCAATAGCGGCAAGTAG-3’; **Pry2** 5’-CTTGCCGACGGGACCACCTTATGTTATT-3’; **LA** 5’-AAATAGATCTCTGGGCTTTTGTTGGTTT-3’; **Plac1Lw1** 5’-GCCTAAATGCGATACCTAAT-3’, **Pry4** 5’-CAATCATATCGCTGTCTCACTCA-3’, **UB** 5’-AACGCGGCCGCCAATAGCGGCAAGTAG-3’.

The *pol32* ^*R2*^ line contains a 2015 bp deletion (positions 15255302–15257317, FB2012_03, released May 11th, 2012), removing nearly all of the *pol32* coding sequence and the 5' neighboring *l(2)35Cc* gene. The *pol32* ^*NR42*^ line carries about a 14 kb deletion (positions 15241415–15255722) that, in addition to a partial deletion of the *pol32* gene, removes the *dao* and *ZnT35C* genes.

### Fertility tests

Female fertility: to determine the embryonic lethality phase, 4-day-old females of the suitable genotype were crossed to *pol32* ^*NR42*^ homozygous males. Eggs were collected from fertilized females using apple-grape-juice agar plates. The eggs were monitored for several days and any first instar larvae were counted and transferred to fresh culture for further development. All the experiments were at 25°. Male fertility was determined as previously described [34].

### Molecular biology

Unless otherwise noted, standard molecular techniques used are described in [35]. Total RNA was purified from female gonadal tissues using the RNA extraction kit (Qiagen) and samples were incubated with DNase I RNase free (Quiagen) to remove any DNA from the preparation, according to manufacturer’s instructions. RT-PCR was done with M-MLV reverse transcriptase using conditions suggested by the suppliers (Invitrogen). For the first-strand cDNA synthesis, 5μg of total RNA were used as a template for oligonucleotide dT primed reverse transcription using SuperScript III RNaseH-reverse transcriptase (Invitrogen), according to manufacturer’s instructions. qRT–PCR was performed in the SmartCycler Real-time PCR (Cepheid) using SYBR green (Celbio) according to the manufacturer’s protocol. For quantification of the transcripts we used the 2ΔΔC_t_ method [36]. The mean of the fold changes and the standard deviation were calculated in three independent experiments.

Primers used in the experiments: **rdU1** Upper Primer 5'-CAGCATTTGGAGGTG AAGTT-3' (position 384 *pol32* cDNA); **rdL1** Lower Primer 5'-CGACTTTGCTGG CTCTGATT-3' (position 568 *pol32* cDNA); **rp49** Upper Primer 5'–ATCGGTTACGGATCGAACAA-3'; **rp49** Lower Primer 5'-GACAATCTCCTTGCGCTTCT-3'.

### Embryo fixation and staining

0–4 h embryos were collected and fixed as previously described [37]; chromatin was visualized by staining with 4,6-diamino-2-phenylindole (DAPI) and images acquired using an epifluorescence microscope equipped with a cooled CCD camera.

### Chromosome cytology

Colchicine-treated metaphase chromosome preparations stained with 4',6-diamidino-2-phenylindole (DAPI) from larval brains were prepared and analyzed as previously described [38]. Brains were dissected in 0.7% sodium chloride, incubated with 10^−5^ M colchicine in 0.7% sodium chloride for 1 h, and treated with hypotonic solution (0.5% sodium citrate) for 7 min. Mitotic chromosome preparations were analyzed using a Zeiss Axioplan epifluorescence microscope (Carl Zeiss, Obezkochen, Germany) equipped with a cooled CCD camera (Photometrics Inc., Woburn, MA).

### Radiosensitivity assay

X-irradiation (2.5 Gy) was performed using a Gilardoni X-ray generator operating at 250 kV and 6 mA at a dose rate of 0.75 Gy/min. Cytological analysis from each genotype was set up in duplicate, one for the analysis of spontaneous chromatid breaks as a control, and another for X-ray treatment. Third instar larvae were irradiated with a dose of 2.5 Gy (corresponding to 250 rad) of X-rays generated by a Gilardoni apparatus (mod. MLG 300/6-D; 250 kV, 6 mA, 0.2 mm copper; Gilardoni, Lecco, Italy). Following a 3 h recovery time, brains were dissected and treated as previously described for cytological analysis. We assessed the presence of chromatid breaks (CD), which are defined as chromatid discontinuities with displacement of the broken segment, and the presence of isochromatid breaks (ISO), defined as two chromatid breaks regarding two different chromatids of the same chromosome.

### Wing Spot Assay

To evaluate the rate of DNA breakage and/or mitotic recombination in null *pol32* flies, we used the Drosophila Somatic Mutation and Recombination Test (SMART). Wings were dissected from flies and analyzed under a compound microscope at 400x magnification. The frequency of spots is calculated as the number of observed spots divided by the number of wings analyzed. Chi-square analysis was used to statistically analyze SMART test data; for Chi-square value calculation, large spots (due to their very low number) were added to the small spots.

### Mutagen treatment

Five-day-old *pol32* ^*NR42*^/*Cy* females were crossed to *pol32* ^*R2*^ */Cy* males for 3 days at 25°. The eggs were allowed to hatch for a further 24 h and then 500 μl of the mutagens EMS (SIGMA CAS No. 62-50-0) or ENU (Sigma-CAS No. 759-73-9), at proper molarity in 5% sucrose, was added to the food. Surviving heterozygous and homozygous adult flies were recorded; a ratio was calculated by dividing the number of *pol32* ^*NR42*^ */pol32* ^*R2*^ flies (phenotypically *Cy* ^*+*^) by the number of heterozygous *pol32* ^*NR42*^ */Cy* and *pol32* ^*R2*^ */Cy* flies (phenotypically *Cy)* at various mutagen concentrations.

### PEV and eye pigment quantification

The effect of *pol32* alleles on variegation of *In(1)w* ^*m4h*^ chromosome was analyzed by crossing *C(1;Y)3*, *In(1)FM7*, *w* ^*1*^ *m* ^*2*^ *B/In(1)w* ^*m4h*^ *; pol32* ^*NR42*^ */In(2LR)Gla* females to 3 types of males without a free Y chromosome: either (i) *C(1;Y)3*, *In(1)FM7*, *w* ^*1*^ *m* ^*2*^ *B/0; pol32* ^*R2*^ */Sco*, (ii) *C(1;Y)3*, *In(1)FM7*, *w* ^*1*^ *m* ^*2*^ *B/0; pol32* ^*rds*^/*Sco* or (iii) *C(1;Y)3*, *In(1)FM7*, *w* ^*1*^ *m* ^*2*^ *B/0; pol32* ^*rd1*^ */Sco*, and 3 types of males with a free Y chromosome: either (iv) *C(1;Y)3*, *In(1)FM7*, *w* ^*1*^ *m* ^*2*^ *B/Y; pol32* ^*R2*^ */Sco*, (v) *C(1;Y)3*, *In(1)FM7*, *w* ^*1*^ *m* ^*2*^ *B/Y; pol32* ^*rds*^/*Sco* or (vi) *C(1;Y)3*, *In(1)FM7*, *w* ^*1*^ *m* ^*2*^ *B/Y; pol32* ^*rd1*^ */Sco*. This allows us to recover male offspring with identical genotypes, differing only in whether they have a Y chromosome, to monitor the effect of the Y on the extent of variegation. All crosses were performed at 25° to prevent temperature effects on variegation. Extraction of the eye pigments and measurement were done according to [39]. Newly hatched males were aged for 6–9 days and 15 excised heads were placed into 1 ml of 30% ethanol acidified to pH 2 with HCl for 3 days. Each sample was split into two tubes for duplicate processing and optical absorbance was measured at 480nm. The two readings for each sample were averaged. Measurement for each line contained from 4 to 12 replicates. Standard deviation was calculated for the values obtained.

To test whether the *pol32* mutation affects TPE, we used the *yw; p[w* ^*+*^ *]39C-58*, and *yw; p[w* ^*+*^ *]39C-50* reporter strains with mini-*w* ^*+*^ inserted near the telomere of 2R *(T2R)*, and *yw; p[w* ^*+*^ *]39-C5* with mini-*w* ^*+*^ insertion in 2L *(T2L)*, as well as the *yw; p[w* ^*+*^ *]39C-31* and *yw; p[w* ^*+*^ *]39C-62* strains carrying mini-*w* ^*+*^ in 3R *(T3R)* [40,41]. Homozygous females from each strain were crossed to *w* ^*1118*^ *; pol32* ^*NR42*^ */Cy* males and the eye phenotypes of the F1 double heterozygous progeny were analyzed. To examine TPE in *pol32* ^*NR42*^ homozygotes, F1 females from each cross were mated to *w* ^*1118*^ *; pol32* ^*NR42*^ */Cy* males. Male and female progeny phenotypically *p[w* ^*+*^ *] pol32* ^*-*^, arising from independent assortment or from exchange events between *pol32* ^*NR42*^ and *T2L p[w* ^*+*^ *]* or *T2R p[w* ^*+*^ *]* chromosomes, were examined for eye pigmentation.

## Results

### New alleles of *pol32*

To obtain null alleles of *pol32* (CG3975), we recovered imprecise excisions of P elements flanking the locus (Fig. 1). *pol32* ^*R2*^ was obtained by the mobilization of P element *P{PZ}ms(2)1006*, which is inserted in the 5’ UTR of *pol32*, while *pol32* ^*NR42*^ comes from excision of the P element *P{EPgy2}CG3975* ^*EY15283*^ inserted downstream of the 3’ UTR of *pol32*.

**Fig 2:**
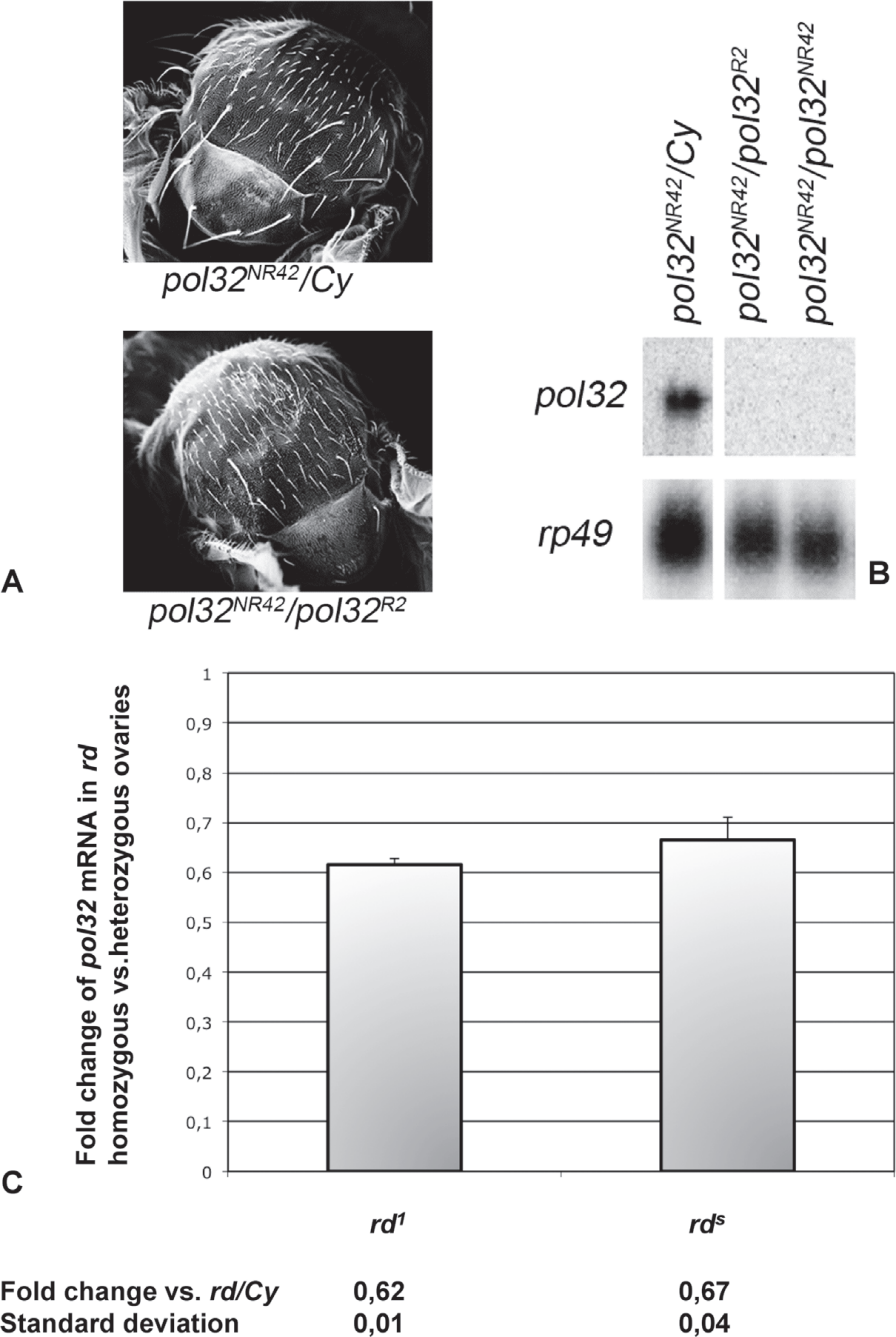
*pol32* expression is altered in *pol32* mutants. (A) Scanning electron micrographs of adult thoraxes: heterozygous *pol32* ^*NR42*^ */Cy* shows a wild-type bristle pattern (upper) while null *pol32* ^*NR42*^ */pol32* ^*R2*^ shows bristles which are shorter or absent (lower). (B) Northern blotting of total RNA extracted from ovaries of the indicated genotypes, hybridized with *pol32* cDNA. A single transcript of about 1.5 Kb is evident in the wild-type ovaries, while no transcript is detectable in the null mutants; hybridization with a ribosomal protein 49 (rp49) cDNA probe is used as a gel-loading control. (C) qRT-PCR analysis on *pol32* transcript in *rd* ^*1*^ and *rd* ^*s*^ homozygous vs. heterozygous. The results come from three independent experiments. Below are indicated the fold change values calculated as reported in Materials and Methods and the standard deviation.

Homozygous *pol32* ^*NR42*^ and *pol32* ^*NR42*^/*pol32* ^*R2*^ trans-heterozygous mutants are female sterile; males are fertile (87 individuals/male; n = 15). Females show short, thin bristles (Fig. 2A) and moderate etching of abdominal tergites; the eggs have apparently normal chorion morphology but don't hatch. Null males have a more severe bristle and abdomen phenotype than females. Molecular characterization of *pol32* ^*R2*^ and *pol32* ^*NR42*^ shows that in both lines the majority of the predicted open reading frame is deleted; *pol32* ^*NR42*^ retains the putative first 180 aa, *pol32* ^*R2*^ retains the last 109 aa. *pol32* ^*R2*^ lethality is due to the deletion of 5' neighboring *l(2)35Cc* gene.

Northern blot analysis shows that the Pol32 transcript of about 1.5 Kb in *pol32* ^*NR42*^ */Cy* ovaries is missing in the *trans*-heterozygous *pol32* ^*NR42*^ */pol32* ^*R2*^ and in the homozygous *pol32* ^*NR42*^ */pol32* ^*NR42*^ ovaries (Fig. 2B).

The physical phenotype reminded us of a mutation called *reduced* (*rd*) [42], described in Lindsley and Zimm [28], located in region 35C3-C5 of the II chromosome [43]. Two alleles, *rd* ^*1*^ and *rd* ^*s*^, are reported to show “bristles reduced in length and thickness, males more extreme than females; males are fertile, but *rd* ^*1*^ females may be sterile” [28]; we refer to this as the *rd* phenotype.

To test whether *pol32* corresponds to *reduced*, we did complementation analysis assessing the *rd* phenotype in *pol32* ^*R2*^ */rd* ^*1*^, *pol32* ^*R2*^ */rd* ^*s*^, *pol32* ^*NR42*^ */rd* ^*1*^ and *pol32* ^*NR42*^ */rd* ^*s*^ trans-heterozygotes. The adults in all genetic combinations show the bristle and the abdomen phenotypes, supporting that *pol32* and *reduced* are synonyms. We then did a molecular characterization of *rd* ^*1*^ and *rd* ^*s*^. The *rd* ^*s*^ mutant contains the transposable element *412*, 4 bp ahead of the translational start codon ATG (Fig. 1). The *rd* ^*1*^ allele contains a DNA insertion of approximately 8 Kb, in the middle of the CDS.

To analyze *pol32* expression pattern in *rd* ^*s*^ and *rd* ^*1*^ mutants, we performed a qRT-PCR on the RNA extracted from homozygous ovaries compared to the heterozygous ovaries. We tested the oligo pairs on the DNA coming from the homozygotes for the two mutations and we obtained amplicons of the right size. Both *rd* ^*s*^ and *rd* ^*1*^ mutants exhibited a reduction (almost 40%) of the *pol32* transcript (Fig. 2C). The difference between the fold change values between the two mutants is not meaningful (p = 0.35 calculated by 2-tailed t-test). Our phenotypic and molecular data identify *rd* mutants as *pol32* alleles; we will henceforth refer to *rd* ^*s*^ and *rd* ^*1*^ as *pol32* ^*rds*^ and *pol32* ^*rd1*^, respectively.

### Pol32 is essential in the early stages of embryogenesis

To investigate the female sterility phenotype, trans-heterozygotes for *pol32* alleles over the deficiency *pol32* ^*R2*^ and in various heteroallelic combinations, were crossed to *pol32* ^*NR42*^ homozygous males (A) or wild-type *Oregon-R* (B) (Table 1). Eggs produced from null *pol32* females, *pol32* ^*R2*^ */pol32* ^*NR42*^ (Cross 1A), don’t hatch and the lethality is not rescued by the paternal *pol32* ^*+*^ allele (Cross 1B). Eggs from *pol32* ^*R2*^/*pol32* ^*rd1*^ and *pol32* ^*R2*^ */pol32* ^*rds*^ females (crosses 2A and 3A, respectively), can infrequently escape early embryonic lethality. *pol32* ^*R2*^ */pol32* ^*rd1*^ embryos reach the larval and pupal stages with very few individuals reaching the adult stage; *pol32* ^*R2*^ */pol32* ^*rds*^ individuals show an earlier lethality and mostly die as larvae. However, the nearly complete rescue of the embryonic lethal phenotype by wild-type paternal allele (crosses B), indicates that both alleles are partial loss-of-function alleles, *pol32* ^*rds*^ being more severe than *pol32* ^*rd1*^. Maternal *pol32* product is critical in the early stages of embryogenesis and a low level of it is sufficient to reach the zygotic stage, when zygotic genes are activated, but not enough to complete development.

**Table 1:** Embryonic lethal effect of *pol32* mutations.

			% of total progeny surviving			
Crosses	Maternal genotype	No. of examined embryos	Hatched	III Instar larvae	Pupae	Adults	
1A	*pol32* ^*R2*^ */pol32* ^*NR42*^	1032	0				
2A	*pol32* ^*R2*^ */pol32* ^*rd1*^	745	11.9	6.6	5.9	0.7	
3A	*pol32* ^*R2*^ */pol32* ^*rds*^	1112	6.7	0.3	0.02	0	
4A	*pol32* ^*rd1*^ */pol32* ^*rds*^	366	12.8	6.3	5.2	2.5	
1B	*pol32* ^*R2*^ */pol32* ^*NR42*^	761	0				
2B	*pol32* ^*R2*^ */pol32* ^*rd1*^	155	80.6	71.6	64.5	41.9	
3B	*pol32* ^*R2*^ */pol32* ^*rds*^	166	80.7	70.5	58.4	44.0	
4B	*pol32* ^*rd1*^ */pol32* ^*rds*^	235	87.7	81.3	78.7	65.5	

Males in A crosses are *pol32* ^*NR42*^ homozygous, in B crosses are wild-type *Oregon-R*.

A low percentage of embryos from *pol32* ^*R2*^ */pol32* ^*rds*^ and *pol32* ^*R2*^ */pol32* ^*rd1*^ mothers are able to hatch and the hatching frequency is increased when these females are crossed to wild-type males. This suggests a zygotic role for Pol32 during embryogenesis.

To test if the arrest of the developing embryos was due to the inability of oocytes to repair DSBs during meiotic recombination in the absence of Pol32, we created double mutant *pol32 mei-W68* flies. *Drosophila mei-W68* mutants lack meiotic DSBs [28], so double mutants should overcome the arrest in embryogenesis. We compared the embryos produced by *pol32* ^*R2*^ */pol32* ^*NR42*^, and *pol32* ^*R2*^ *mei-W68/ pol32* ^*NR42*^ *mei-W68* homozygous mothers.

Embryos were collected during 0–4 h time period, fixed and stained with DAPI to visualize the nuclei. Essentially, we can classify the mutant embryos into 3 phenotypic classes, and between single and double mutant embryos there is no significant change in the frequencies and in the types of the observed phenotypes (Table 2 and S1 Fig.). Approximately 11% of the *pol32* ^*-*^ mutant embryos show a polar body with a star like structure with additional or fragmented chromosomes; about 57% of the embryos arrest in mitotic cycles 1 to 6 of with uneven distribution of nuclei; the remaining 31% of embryos, that we define as degenerated, show bubble-like formations and no chromatin is detectable (S1 Fig.). In the mutant embryos we count no more than 64 nuclei and no embryo forms polar cells. When 0–4 h mutant embryos are aged for an additional 4 h all embryos appear degenerated. To see if embryo degeneration is an artifact of the collection time period, we decreased this interval to 1 h. The percentage of degenerated embryos decreased by up to a third, suggesting that about 20% of the embryos observed in the 0–4 h collection degenerate after nuclear division arrest, while another 10% could degenerate due to defects during oogenesis.

**Table 2:** Frequencies of embryonic phenotypes observed in 0–4 h collection from *pol32* ^*R2*^ */pol32* ^*NR42*^ and *pol32* ^*R2*^ *mei-W68/pol32* ^*NR42*^ *mei-W68* mothers.

**Maternal genotype**	**1 nucleus (%)**	**1–6 nuclear division (%)**	**7–16 nuclear divisions(%)**	**Degenerate (%)**	**No. of counted embryos**
*Oregon-R*	2	10	88	0	200
*pol32* ^*R2*^ */pol32* ^*NR42*^	11.4	57.3	0	31.2	420
*pol32* ^*R2*^ *mei-W68/ pol32* ^*NR42*^ *mei-W68*	9.2	61.7	0	29.2	379

Pol32 functions as a subunit of both replicative and TLS pols, but the defects exhibited by the *pol32* mutant embryos seem to be essentially due to the role of Pol32 as a subunit of replicative Polδ as suggested by Rong [13]. These mutants prevent the correct replication of DNA during the fast syncytial nuclei divisions of the embryos.

### Pol32 is required for chromosome stability

#### a. X-ray sensitivity

**Fig 3:**
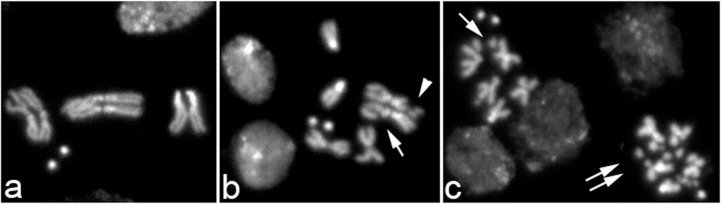
Mutations in *pol32* cause chromosome breakage. Representative panels of metaphases from (a) wild-type and (b, c) *pol32* ^*NR42*^ */pol32* ^R2^ brains, showing CD (arrow), ISO breaks (head arrow) and extensive chromosome fragmentation (double arrows).

Although null *pol32* homozygous flies are fully viable, colchicine-treated larval brain squashes from *pol32* null mutants revealed a high frequency of spontaneous chromosome breakage. Of the mitotic *pol32* ^*-*^ cells, 5.5% displayed chromosome breaks, whilst the frequency in control cells was approximately 0.3% (Table 3). These breaks involve either one chromatid (CD) or both sister chromatids (isochromatid break, ISO) and are characterized by the simultaneous presence of both the acentric and the centric fragments (Fig. 3).

**Table 3:** Frequencies of chromatid (CD) and isochromatid (ISO) breaks observed in *pol32* null mutant brains.

**IR treatment(Gy)**	**Genotype**	**No. of brains**	**No. of scoredcells**	**% of cells with breaks**	**% of cells with >5 breaks per cell**
0	*+/+*	6	618	0.3	0
	*pol32* ^*NR42*^ */pol32* ^*R2*^	8	1906	5.5	0
2.5	*+/+*	4	394	8.9	0
	*pol32* ^*NR42*^ */pol32* ^*R2*^	4	488	58.5	1.8

To substantiate the finding that Pol32 is required to prevent chromosome breakage, we treated mutant and control larvae with X-rays. Third instar larvae were irradiated with 2.5 Gy and after a 3 h recovery, brains were dissected and then incubated in colchicine for 1 h before fixation. The results show that *pol32* mutant cells are considerably more sensitive than wild-type cells to the induction of chromosome breaks (Table 3). 58.5% of the mitotic brain cells from irradiated *pol32* mutant larvae displayed chromosome breaks, compared to the 8.9% of chromosome breaks induced in control brains. Moreover, in 1.8% of the aberrant metaphases from treated *pol32* larvae, we detected extensive chromosome fragmentation (more than 5 chromosome breaks per cell), which is not present in treated control cells (Fig. 3). Thus, *pol32* function is essential both for preventing spontaneous chromosome breakage and for repairing IR-induced DNA damage.

#### b. mitotic chromosome breakage

The Drosophila Somatic Mutation and Recombination Test (SMART), also known as the Wing Spot Assay, is routinely used for a qualitative and quantitative evaluation of genetic alteration induced by mutants defective in DNA repair. It detects a loss of heterozygosity (LOH) resulting from gene mutation, chromosome rearrangement, chromosome breakage and mitotic recombination [44].

SMART employs the wing cell recessive markers *multiple wing hairs* (*mwh*, 3–0.7) and *flare* ^*3*^ (*flr* ^*3*^, 3–38.8) in transheterozygous *mwh +/+ flr* ^*3*^ individuals. When a genetic alteration is induced in a mitotically dividing cell of a developing wing disc, it may give rise to a small (1–2 cells) or large clone(s) (> 3 cells) of mutant *mwh* and/or *flr* ^*3*^ cells (a “spot”) visible on the wing surface of the adult fly. Two types of spots can be produced: (i) single *mwh* or *flr* ^*3*^ spots and (ii) twin *mwh* and *flr* ^*3*^ spots (patches of adjacent *flr* ^*3*^ and *mwh* cells). The types of clone can reveal the mutational mechanisms involved in clone production; single spots are produced by somatic point mutations, chromosome aberrations or mitotic recombination; twin spots originate exclusively from mitotic recombination. We used the SMART test to evaluate the rate of DNA breakage and/or mitotic recombination in null *pol32* flies. We compared the frequencies of single spots and twin spots observed using the two genotypes *mwh/TM6* and *mwh +/+ flr* ^*3*^, in wild-type and *pol32* ^*-*^ backgrounds (Table 4). Males *pol32* ^*NR42*^ */Cy; mwh jv/TM6*, *Tb e* were crossed to *pol32* ^*R2*^ */Cy; mwh jv/TM6*, *Tb e* females and wing blades of *pol32* ^*R2*^ */pol32* ^*NR42*^ *; mwh/TM6*, *Tb e* individuals were scored for *mwh* single spots arising in cells rendered hemizygous or homozygous due to a deletion or mutation event; mitotic recombination products between the balancer chromosome *TM6* and the normal *mwh* chromosome are inviable.

**Table 4:** Wing spot test data in *pol32* null mutant.

**Genotypes**	**Total wings**	**Spots per fly**							
		**Small single spots**		**Large single spots**		**Small twin spots**		**Large twin spots**	
		**(1–2 cells)**		**(>2 cells)**		**(1–2 cells)**		**(>2 cells)**	
		**Fr.**	**No.**	**Fr.**	**No.**	**Fr.**	**No.**	**Fr.**	**No.**
*+/+; mwh/TM6*	108	0.01	1		0		0		0
*pol32* ^*R2*^ */pol32* ^*NR42*^ *; mwh/TM6*	94	1.0	98	0.02	2		0		0
*+/+; mwh +/+ flr* ^*3*^	80		0		0		0		0
*pol32* ^*R2*^ */pol32* ^*NR42*^ *; mwh +/+ flr* ^*3*^	120	0.9	105	0.05	6	0.01	1	0.03	4

Fr., frequency of spots calculated as the number of observed spots divided by the number of wings analyzed.

No., number of spots.

Males *pol32* ^*NR42*^ */Cy; mwh jv/TM6*, *Tb e* were crossed to *pol32* ^*R2*^ */Cy; flr3/TM6*, *Tb e*. Among the progeny, individuals *pol32* ^*R2*^ */pol32* ^*NR42*^ *; mwh +/+ flr* ^*3*^ can produce single and twin spots. Twin spots are exclusively derived from mitotic recombination. Both *pol32* ^*R2*^ */pol32* ^*NR42*^ *; mwh/TM6* and *pol32* ^*R2*^ */pol32* ^*NR42*^ *; mwh +/+ flr* ^*3*^ progeny produce single spots, with the mean frequency of the *mwh* or *mwh/flr* ^*3*^ spots per wing of approximately 1.0 in both genotypes (Table 4). There is a significant effect of the *pol32* mutation on the production of single spots (Chi-square = 189.12, df = 2, p<1E-10). The very low number of twin spots compared to single spots in null *pol32* flies indicates that almost all of the events arise not from recombination, but from chromosome breaks. These results confirm the participation of Pol32 in the repair of DNA lesions that give rise to chromosome breakage.

#### c. sensitivity to monoalkylating agents

**Fig 4:**
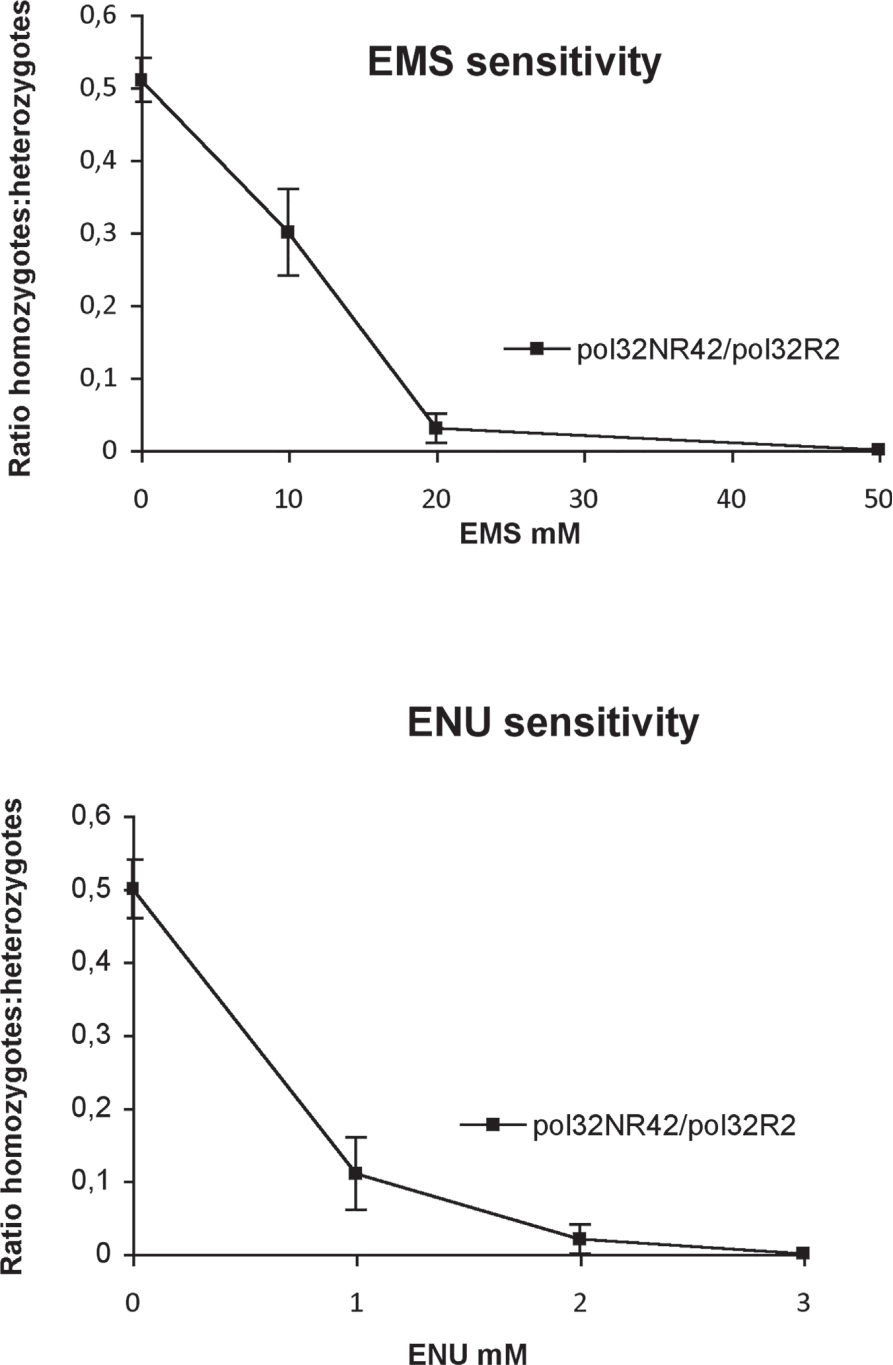
*pol32* null mutants are sensitive to EMS and ENU. Newly- hatched larvae from a cross of *pol32* ^*NR42*^ */Cy* X *pol32* ^*R2*^/*Cy* were treated with EMS and ENU at various concentrations; the ratio of homozygous *pol32* ^*NR42*^ */ pol32* ^*R2*^: heterozygous *pol32* ^*-*^ */Cy* surviving adults was plotted against mutagen concentration. The lower frequency of *pol32* ^*NR42*^ */pol32* ^*R2*^ homozygous flies observed at increasing mutagen doses demonstrates the mutant hypersensitivity to EMS and ENU. Error bars correspond to Standard Deviation determined from at least five independent experiments.

We tested the sensitivity of *pol32* null mutant to the monoalkylating agents ethylmethanesulphonate (EMS) and N-ethyl-N-nitrosourea (ENU) at increasing concentrations. We know that *pol32* mutants are extremely sensitive to MMS [13], an alkylating agent inducing mainly chromosome aberrations [45]. Due to their different reactive mechanisms, the repair of DNA alterations induced by MMS, EMS and ENU mutagens requires various DNA repair processes [46–48]. Larvae from a cross of *pol32* ^*NR42*^ */Cy* females by *pol32* ^*R2*^ */Cy* males were treated with EMS and ENU, and the adult flies were scored for heterozygosity or homozygosity. The results (Fig. 4 and S1 Table) indicate a significant loss of viability of homozygous *pol32* ^*R2*^ */pol32* ^*NR42*^ flies compared to their heterozygous siblings, at all concentrations of the tested mutagens, with total lethality at 50mM EMS and 3mM ENU. Thus *pol32* ^−^flies are hypersensitive to both mutagens, suggesting the participation of Pol32 in the repair pathways triggered by these monoalkylating agents.

### *pol32* mutants suppress Position Effect Variegation

Chromatin modifications are necessary for the recognition of DNA lesions and for access to various protein complexes during DNA repair [49–50]. A role for epigenetic changes in a chromatin-dependent repair mechanism in DSB repair is now emerging [51]. DNA repair-deficient mutants (e.g. *mus* genes) are potential candidates for modifiers of position effect variegation (PEV) [52–53], so we looked at whether Pol32 is a PEV modifier.

PEV is an epigenetic phenotype produced by the inactivation of a euchromatic gene when relocated in, or close to, the heterochromatin [54]. PEV may be altered by suppressor or enhancer mutations [55]. Variegation is also suppressed by the addition of extra heterochromatin, such as a Y chromosome, and enhanced by decreasing the heterochromatin, which occurs in *X/0* males [56].

**Fig 5:**
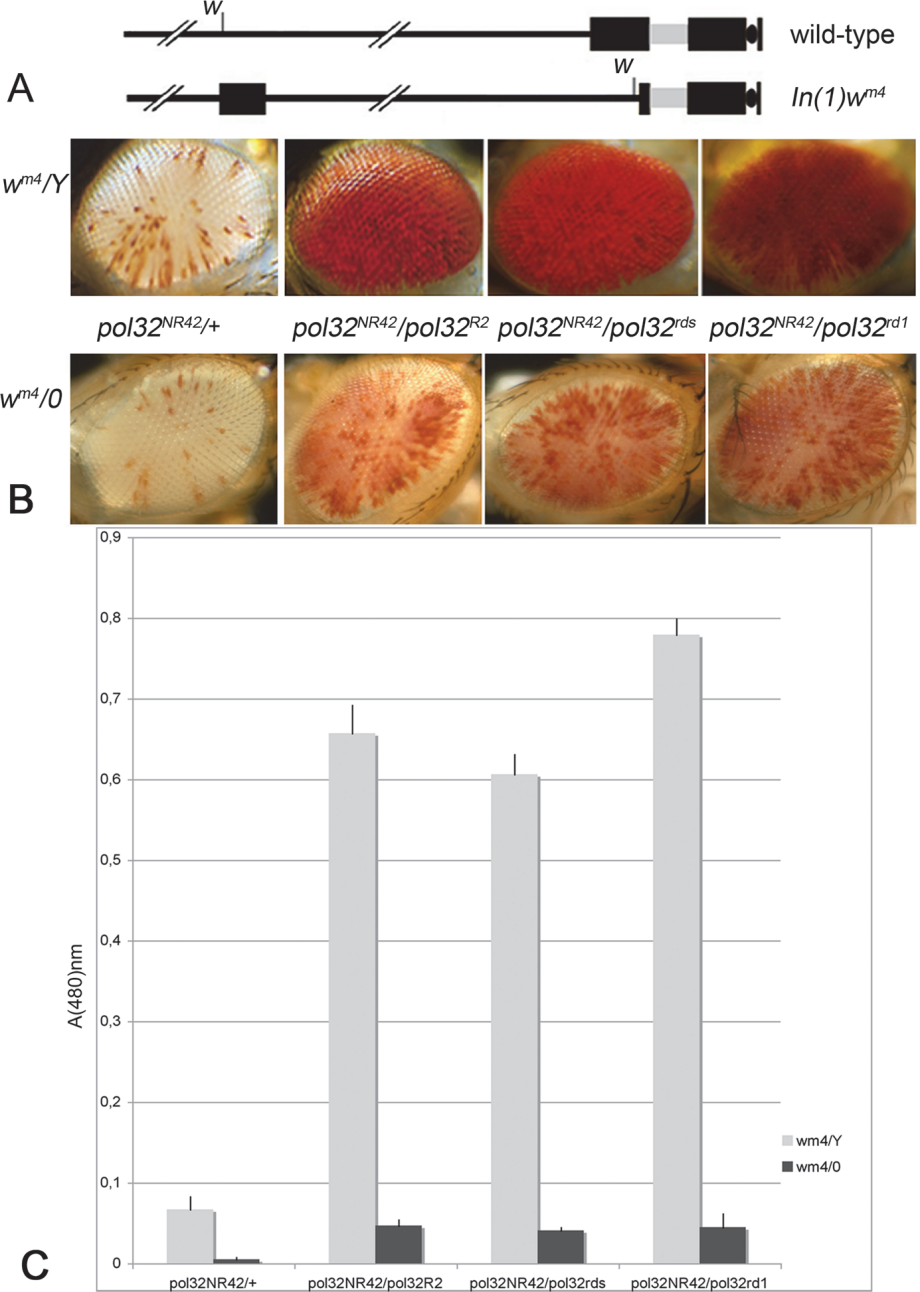
Mutations in *pol32* suppress PEV. (A) Diagram of *In(1)w* ^*m4*^ chromosome. The black boxes represent heterochromatic regions, the gray box represents the heterochromatic *bb* locus. (B) Phenotypic effect of *pol32* mutations on the expression of *white (w)* in *In(1)w* ^*m4*^ males of the indicated genotypes. Inactivation of *w* is seen in eyes with only rare and small patches of red pigment. The PEV suppression of the Y chromosome, enhances pigmentation in *X/Y* with respect to *X/0* males. *w* expression is enhanced in all *pol32* allelic combinations. (C) Spectrophotometric quantitation of eye pigment. Error bars represent the standard error.

We used the inversion chromosome, *In(1)w* ^*m4h*^ (referred to here as *w* ^*m4*^) in which the *white (w)* gene is moved close to the basal heterochromatin of the X chromosome (Fig. 5A), and the new position induces a variegated pigment phenotype in the eyes. The amount of eye pigment is diagnostic of the strength of PEV. Individuals carrying *pol32* alleles in various genetic combinations were analyzed for variegation of *w* in the *w* ^*m4*^ chromosome. Fig. 5B displays the expression of *w* both in the presence and in the absence of the Y chromosome in *pol32* ^*-*^ males. The strong inactivation of *w* in *w* ^*m4*^/*Y* males leaves only rare and small dots of red pigment in the eyes; in *w* ^*m4*^/*0* males the eyes appear quite white. In the state of partial (*pol32* ^*NR42*^/*pol32* ^*rds*^ and *pol32* ^*NR42*^/*pol32* ^*rd1*^) or complete (*pol32* ^*NR42*^ */pol32* ^*R2*^) loss of Pol32, mottling of the phenotype increases in *w* ^*m4*^/*0* males, and *w* ^*m4*^/*Y* males are almost fully pigmented. Spectrophotometrical quantization of the pigment level confirms a correlation between the eye phenotype and the amount of drosopterin (Fig. 5C).

PEV may also occur on genes inserted into the telomeres, a silencing phenomenon called telomere position effect (TPE) [57,58]. We tested the effect of null *pol32* mutation on TPE, using a mini-*white* ^*+*^ reporter gene inserted into telomere-associated sequences (described in Materials and Methods). Analyzing eye pigmentation in both heterozygous and homozygous *pol32* ^*NR42*^ flies carrying the *w* ^*+*^ reporter, we found no differences compared to controls. This is not surprising: systematic searches for TPE and PEV modifiers reveal that the majority of mutations that affect PEV have no effect on TPE, suggesting that PEV and TPE differ mechanistically [41,58]. The *pol32* mutation suppresses PEV and does not affect TPE.

## Discussion

### *pol32*, DNA replication and repair

The Pol32 protein is involved in several processes that promote genome stability, which can be seen from many studies conducted in yeast. By analyzing *Drosophila* phenotypes in null or hypomorph *pol32* mutants, we defined the specific processes in which Pol32 is required. It has an essential function in DNA replication during the early embryonic divisions, corresponding to the period in which each nucleus replicates its DNA and divides in less than 10 minutes. The small percentage of embryos that we define as degenerated, together with the embryos showing a polar body-like structure, may also indicate a role for Pol32 during oogenesis. Cytogenetic analysis shows that chromosome breaks characterize the *pol32* mutant and that the mutants have an increased sensitivity to X-rays, providing evidence that Pol32 ensures chromosome integrity in somatic cells. The high frequency of single spots with respect to twin spots in the SMART test also supports a Pol32 role in the repair of DNA lesions that cause chromosome breaks. The increased sensitivity of *pol32* mutants to the monoalkylating agents ENU and EMS, as well as MMS, suggests the involvement of Pol32 in diverse DNA repair pathways.

In an RNAi-based screen to identify MMS survival genes in *D*.*melanogaster*, the analysis of the protein interactome placed Pol32 (as CG3975) in BER (Base Excision Repair), NER (Nucleotide Excision Repair) and TOR (Target of Rapamycin) pathways [12].

In mammalian cells, BER—through DNA polymerase β (Polβ), Polδ and Polε—is the main pathway that cells use to repair abasic (AP) sites [59,60]. In *D*. *melanogaster*, which lacks the Polβ ortholog, the involvement of DmPolζ has been suggested in AP site repair [48]. Since the deletion of Pol32 leaves both Polδ and Polζ structurally incomplete, analysis of *pol32* mutants combined with knockout in the single subunits of these Pols and of the enzymes involved in the other pathways will clarify the relationship of Pol32 in the DNA damage survival network.

### pol32 and PEV

We found that *pol32* mutations suppress PEV in the *w* ^*m4*^ chromosome. This suggests an involvement of Pol32 in the induction of chromatin state changes. A switching role that permits the replacement of Polδ with Polζ and *vice versa* has been proposed in yeast for the complex of Pol32 and Pol31. This accessory protein complex, retained on DNA at replication-blocking lesions, exchanges Polδ with Polζ during TLS DNA synthesis, allowing replication to proceed [2–4]. The mechanism by which these events occur is unknown, but they require the interaction of Pol32-Pol31, through Pol32, with the processivity factor PCNA.

Chromatin modifications occur during the DNA damage response and Pol32 could enter in this process. An involvement of Pol32 in the induction of chromatin state changes may include both its activities, DNA replication and repair.

The abnormal bristles and tergites phenotype, observed in *pol32* ^*-*^ mutants, recalls *bobbed (bb)* mutants, which are caused by a reduction in rRNA synthesis [61]. Investigations are required to determine whether Pol32 can affect the rDNA repeat number and/or the chromatin structure of this region. Genetic analyses have demonstrated the influence of some repair defective mutations on the chromosomal stability of the rDNA cluster [62,63]. An effect of Pol32 on rDNA repeat expansion has been seen in yeast [64]. Human p66, the ortholog of Pol32, interacts directly with WERNER protein (WRN); in cells from individuals with a deficiency in WRN helicase, rDNA gene arrays display an increased proportion of palindromic structures [65,66]. Finding an evolutionary conservation of functional interactions of p66 with WRN and with components of the DNA repair pathway may render *Drosophila* an interesting model for studying relationships among genes associated with genomic instability.

## Supporting Information

S1 Fig Altered development of *pol32* null embryos. Embryos collected at 0–4 h from wild-type *Oregon-R* (a) and *pol32* ^*R2*^ */ pol32* ^*NR42*^ (b-e) mothers, stained with DAPI and viewed as whole mounts. (a) Wild-type embryo at blastoderm stage: nuclei are uniformly distributed along the cortex; in the middle of the embryo dividing nuclei are visible; pole cells are visible at the posterior end. (b) *pol32* ^*R2*^ */pol32* ^*NR42*^ embryo showing asynchronous nuclear divisions, abnormal spatial arrangements of syncytial nuclei; the difference in intensity of staining suggests a different level of ploidy. Mutant embryos at higher magnification show chromatin fragmentation (c), metaphase-anaphase with dispersed chromosomes and chromatin (d), anaphase chromosome bridges (e).
(TIF)

S1 Table Survival to adulthood after EMS or ENU treatment. (DOC)
