# Supplementary figures and images for: Loss of Pol32 in Drosophila melanogaster Causes Chromosome Instability and Suppresses Variegation
Source: PLoS One. 2015 Mar 31;10(3):e0120859. doi: 10.1371/journal.pone.0120859 (PMC4380491; doi:10.1371/journal.pone.0120859)

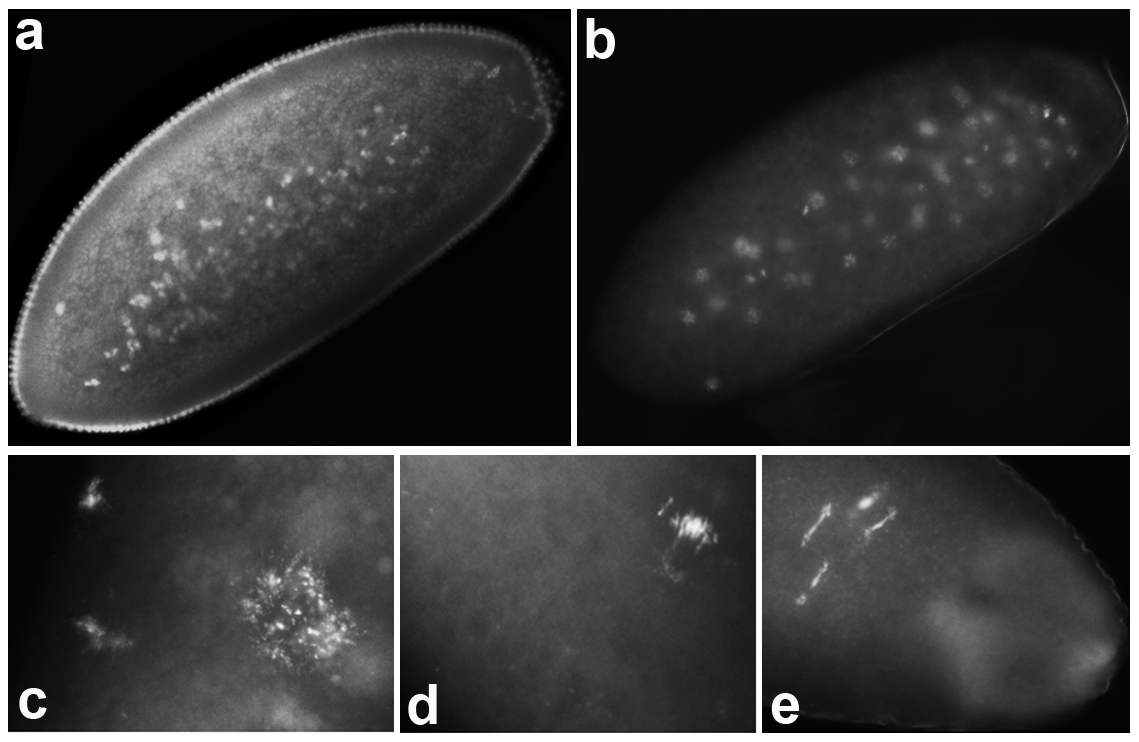

Supplement: S1 Fig — Embryos collected at 0–4 h from wild-type Oregon-R (a) and pol32 R2 / pol32 NR42 (b-e) mothers, stained with DAPI and viewed as whole mounts. (a) Wild-type embryo at blastoderm stage: nuclei are uniformly distributed along the cortex; in the middle of the embryo dividing nuclei are visible; pole cells are visible at the posterior end. (b) pol32 R2 /pol32 NR42 embryo showing asynchronous nuclear divisions, abnormal spatial arrangements of syncytial nuclei; the difference in intensity of staining suggests a different level of ploidy. Mutant embryos at higher magnification show chromatin fragmentation (c), metaphase-anaphase with dispersed chromosomes and chromatin (d), anaphase chromosome bridges (e). (TIF) [file pone.0120859.s001.tif]
