# Supplementary material for: Loss of Pol32 in Drosophila melanogaster Causes Chromosome Instability and Suppresses Variegation
Source: PLoS One. 2015 Mar 31;10(3):e0120859. doi: 10.1371/journal.pone.0120859 (PMC4380491; doi:10.1371/journal.pone.0120859)
Supplement: S1 Table — (DOC) [file pone.0120859.s002.doc]

**S1 Table. Survival to adulthood after EMS or ENU treatment.**

| Crosses  *pol32^NR42^/Cy x pol32^R2^/Cy* | | | |
| --- | --- | --- | --- |
|  | No. of flies^a^ | | Ratio^b^ |
|  | *pol32^-^/Cy* | *pol32^NR42^/pol32^R2^* |  |
| **EMS** |  |  |  |
| 0 mM | 1063 | 546 | 0.51 |
| 10 mM | 570 | 170 | 0.30 |
| 20mM | 678 | 22 | 0.03 |
| 50 mM | 1002 | 0 | 0 |
| **ENU** |  | | |
| 0 mM | 1557 | 778 | 0.5 |
| 1 mM | 687 | 75 | 0.11 |
| 2 mM | 917 | 21 | 0.02 |
| 3 mM | 581 | 0 | 0 |

^a^ Adult flies recovered after treatment with specified mutagen at the

indicated concentrations.

^b^ Number of *pol32^NR42^/pol32^R2^* flies divided by the number of

heterozygous *pol32^NR42^/Cy* and *pol32^R2^/Cy* flies.
